# Perimenopause and/or menopause help-seeking among women from ethnic minorities: a qualitative study of primary care practitioners’ experiences

**DOI:** 10.3399/BJGP.2022.0569

**Published:** 2023-05-03

**Authors:** Jennifer MacLellan, Sharon Dixon, Sultana Bi, Francine Toye, Abigail McNiven

**Affiliations:** Nuffield Department of Primary Care Health Sciences, University of Oxford, UK.; Nuffield Department of Primary Care Health Sciences, University of Oxford, UK.; The Lister Surgery, Westbourne Green Community Health Centre, Bradford.; Physiotherapy Research Unit, Oxford University Hospitals NHS Foundation Trust, UK.; Nuffield Department of Primary Care Health Sciences, University of Oxford, UK.

**Keywords:** communication, ethnic minority, menopause, perimenopause, primary health care

## Abstract

**Background:**

Each woman’s experience of the perimenopause and/or menopause is individual and unique. Research shows women from ethnic minorities often have different experiences from their White peers, and these are not being considered in conversations about the menopause. Women from ethnic minorities already face barriers to help-seeking in primary care, and clinicians have expressed challenges in cross cultural communication including the risk that women from ethnic minorities’ perimenopause and/or menopause health needs are not being met.

**Aim:**

To explore primary care practitioners’ experiences of perimenopause and/or menopause help-seeking among women from ethnic minorities.

**Design and setting:**

A qualitative study of 46 primary care practitioners from 35 practices across 5 regions of England, with patient and public involvement (PPI) consultations with 14 women from three ethnic minority groups.

**Method:**

Primary care practitioners were surveyed using an exploratory approach. Online and telephone interviews were conducted and the data were analysed thematically. The findings were presented to three groups of women from ethnic minorities to inform interpretation of the data.

**Results:**

Practitioners described a lack of awareness of perimenopause and/or menopause among many women from ethnic minorities, which they felt impacted their help-seeking and communication of symptoms. Cultural expressions of embodied experiences could offer challenges to practitioners to ‘join the dots’ and interpret experiences through a holistic menopause care lens. Feedback from the women from ethnic minorities provided context to practitioner findings through examples from their individual experiences.

**Conclusion:**

There is a need for increased awareness and trustworthy information resources to help women from ethnic minorities prepare for the menopause, and clinicians to recognise their experiences and offer support. This could improve women’s immediate quality of life and potentially reduce future disease risk.

## INTRODUCTION

The menopause is a natural stage in a woman’s life, and each woman’s experience is individual and unique. Research from the US shows that women from different ethnic groups may have different experiences from their White peers, such as entering the perimenopause and/or menopause at earlier ages,^1^ having longer transition periods,^2^ and an emphasis on different symptoms.^3^^–^5 Acculturation and the stresses of poverty and structural racism appear to play a complex role in menopausal symptomatology, with recommendations to clinicians that ethnicity should be taken into account when interpreting symptom presentation.^6^^–^9 Despite evidence of high menopausal symptom burden among women from ethnic minorities from studies outside the UK, evidence of help-seeking and hormone replacement therapy (HRT) treatment are less common when compared with White women.^10^ In the UK, ethnic minority groups are over represented in the 20% most deprived areas.^11^^,^^12^ Inequalities in HRT prescribing in the UK may reflect an unmet need in menopause care for women living in deprived areas, and requires further investigation.^13^

In the UK, research evidence exploring women from ethnic minorities’ experiences of the menopause is limited^14^ and largely confined to journalism, charities, and advocacy blogs from women in the ethnic minority community.^15^^–^17 These narratives stress how the specific and nuanced aspects of the experiences of women from ethnic minorities are not being considered in the conversations about menopause.^18^^,^^19^ In the UK, the majority of patients with perimenopause and/or menopausal symptomatology will present and be managed in primary care, which is ideally placed for its accessibility and holistic approach.^20^

The literature shows that women from ethnic minorities face numerous barriers to help-seeking in primary care that include language, inhibition, and constraints in health literacy.^21^^,^^22^ Studies exploring the professional perspective focus on the challenges of cross cultural communication.^23^ This study explores primary care practitioners’ experiences of women from ethnic minorities seeking help for perimenopause and/or menopause symptoms. The study findings were explored with groups of women from ethnic minorities to provide context and improve understanding of the results; their responses are presented alongside, and sometimes in juxtaposition, to that of the healthcare practitioners.

**Table:** How this fits in

Women from ethnic minorities may present with symptoms of perimenopause and/or menopause that are different, or described differently, from their White peers. This study has shown a communication gap in the consultation, rising from lack of awareness of perimenopause and/or menopausal symptomatology in some women from ethnic minorities which does not allow them to advocate for their health, compounded by a lack of knowledge or confidence in some primary care clinicians to interpret and connect symptom presentation with a holistic menopause care lens. This can impact women’s quality of life and their future disease risk.

## METHOD

This study was part of a larger qualitative project exploring primary care practitioners’ experiences of supporting women’s health in primary care in England between March and September 2022. As the research question was broad, an exploratory approach was adopted. Sampling was targeted towards primary care practitioners working in areas of deprivation where health inequalities, multimorbidity, and GP retention challenges are keenly felt. Participants answered an invitation, accompanied by an information sheet and consent form, through the Clinical Research Network.

The original topic guide was developed in response to a perceived gap in the women’s health strategy evidence base. The guide was piloted with two practitioners and used to create a broad structure for the interview, with flexibility for the participant to develop and expand on the topics/issues of particular importance or relevance to their context of practice. Data were collected through single episode, one-to-one interviews with fully informed consent recorded, conducted virtually online or by telephone by experienced qualitative researchers. Audio recordings were transcribed verbatim, checked against the original recording, and loaded into the data analysis software, NVivo 12.

The data were analysed thematically as data collection progressed.^24^ The primary researcher initially coded the transcripts, discussing the coding frame and results in regular analysis meetings with the wider research team. Not all study participants had experience of consulting with women from ethnic minorities. However, the specific practice challenges relating to perimenopause and/or menopause symptom help-seeking and healthcare access among women from ethnic minorities began to emerge strongly from the data through the constant comparison of codes and new data.

Practices serving highly diverse populations were purposively sampled to clarify, check, and confirm the findings. Analysis progressed to focused coding and development of core categories by the primary researcher before interpretation into themes in discussion with the full research team. Sampling continued until data saturation was reached, as agreed by the full team.^25^

### Consulting with women from ethnic minorities with lived experience of the menopause

In order to glean real-life experiences and inform interpretation of the data, the findings were presented to three groups of women from ethnic minorities:

- those identifying as Black African;
- those identifying as South Asian from an Urdu speaking community;
- those identifying as South Asian from a Bengali speaking community.

Women were invited to participate in the study through a university ‘Diversity in research’ group and two community-based organisations offering a range of health and wellbeing support to the local community. Three meetings were held lasting between 1.5 to 2.5 hours, with 14 participants in total who had experience of perimenopause and/or menopausal symptoms. One meeting was conducted online in English (with two women), and two meetings were in-person with interpreter support (with 12 women). The in-person meetings included five women who communicated only in their own language with interpreter support, three who spoke a mix of English and their own language, with four communicating primarily in English. All participants spoke the community language (either Bengali for the Bengali-speaking women’s group or Urdu for the Urdu-speaking women’s group) and participated fully in the lively discussions. Interpretation did not disrupt the flow of the discussion but was conducted discreetly for the researcher to keep up with the conversation and ask clarifying questions. Participants were reimbursed for their time. In this article, the women’s perspectives are presented alongside the clinician experience, weaving together the research participants’ and the women’s voices, with recognition that good healthcare must take into account both sets of experiences and address both sets of needs.

## RESULTS

**Figure 1.**
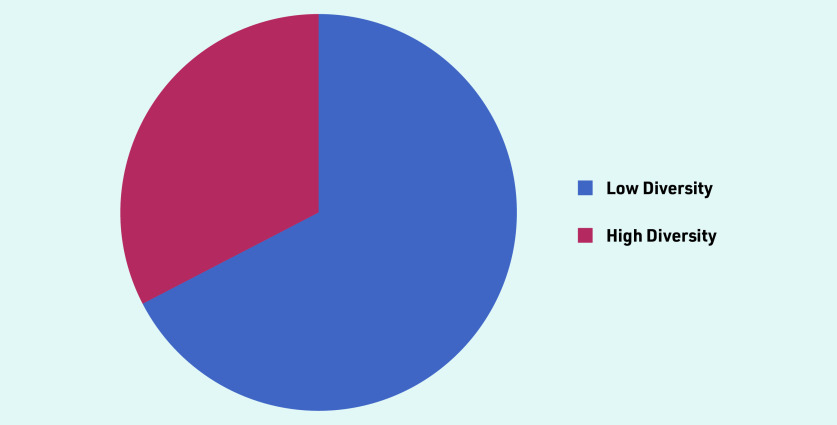
*Population diversity of GP practice postcode. If >86% of the population is White = Low Diversity If <86% of the population is White = High Diversity*

**Figure 2.**
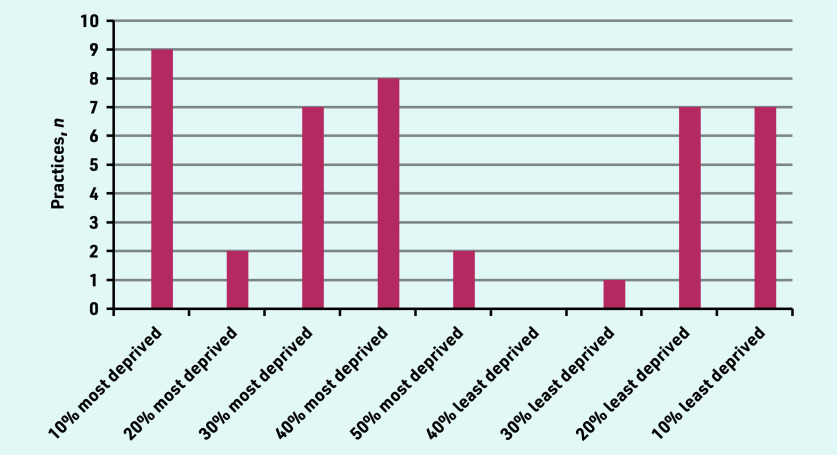
*Multiple deprivation index of practice postcode.*

A total of 46 primary care practitioners were interviewed, from 35 practices across five regions of England, (Central and North West London, Greater Manchester, South London, Thames Valley and South Midlands, Yorkshire and Humber). The deprivation index and ethnic diversity of the practice postcode offer an illustration of the population context (Figures 1 and 2). Participant characteristics are recorded in Table 1. Interviews lasted from 19 to 60 minutes, with an average duration of 32 minutes.

**Table 1.** Participant characteristics

**Role**	**Gender**		**Experience (years) Range (average)**	**Special interest women’s health**
	**Male**	**Female**		
GP	5	26	1–30 (11)	10
Nurse	0	9	3–20 (14)	0
Pharmacist, paramedic, associate	0	6	9–30 (13)	0

The analysis constructed three themes: knowing the problem, communicating the problem, and joining the dots (a holistic interpretation of presentation).

Participants spoke candidly during the interviews with deep respect for patients and colleagues, often identifying challenges as system constraints.

### Knowing the problem

The practitioners interviewed described a significant increase in the overall numbers of women attending consultations looking for support for symptoms of perimenopause and/or menopause in recent years, largely credited to recent campaigns in the media. However, this surge was not perceived to be equally reflected among ethnic minority groups in the population:

*‘I see White women with menopause. There’s so many other ethnicities in* [city in the North of England] *that we don’t see as much of, and I’m s*ure they’re suffering the same symptoms.*’*

(PC08, Clinical Pharmacist, city, North of England)

Cultural sensitivity surrounding the discussion of reproductive health and its embodied experience, within families and the community, was suggested as a factor limiting women’s attribution of their symptoms to the hormonal changes in their body. Consequently, practitioners reported that while women from ethnic minorities often had an awareness of the menopause as the end of a woman’s periods, associated symptoms surrounding the transition to and beyond this life stage were less well understood:

*‘I say “Oh, do you get sweats and things?” and they’ll say, “Oh yes, I do get them,” and I’ll say, “Oh, do you think you might be going through the menopause?” But they’re like, “Oh, but I’m still having periods.” The idea that there might be a perimenopause and actually that this is a transition, I don’t think is something that* [all] *people really particularly* [understand]*.’*

(PC14, GP, city, North of England)

Participants said that many consultations about the menopause involved educating women across population groups about potential symptom presentation, as a prerequisite for treatment or self-care options available to them. Although there has been a welcome increase in the availability of English language resources, a particular constraint mentioned by practitioners was the lack of educational resources in the different languages spoken by their practice populations, and accessible routes of delivery:

*‘We should be producing leaflets about the menopause in Urdu, Punjabi, all these different languages so that women can access that.’*

(PC20, GP, Midlands, rural)

*‘One of my friends, she’s a doctor in a different region where there’s a very large Asian population, and she did this radio show where she would talk about different health concerns* […] *tailored to the population, and she spent quite a lot of time talking about women’s health and periods, menopause, sexual health, contraception, and she said there’s really good engagement.’*

(PC21, GP, city, central England)

Participants felt that the provision of accurate information for women was extremely important to ensure understanding of the symptoms they were experiencing and their relation to the changing hormones in their body. Not knowing that symptoms may be related to perimenopause and/or menopause and that treatment or self-care choices were available to help alleviate symptoms was seen as a barrier to help-seeking for some women.

#### Reflections from the lived experience group

The results were shared with 14 women from ethnic minorities with lived experience of perimenopause and/or menopause, and they were asked for their reflections on how the findings resonated with their perspectives.

The group described the menopause as *‘when your periods stop.’* (comment made by many participants). The majority of the group described how women in their community would feel embarrassed to talk about symptoms, that the menopause is a natural phase of a woman’s life and they need to *‘just get on with it,’* or, *‘if you can manage it, then don’t speak about it.’* (comment made by many participants). One participant observed how some women do not want to accept they are getting older and are no longer able to have children.

When asked how awareness of perimenopause and/or menopause could be addressed in the community, the Bengali women specifically advocated for visual messaging through links to video clips from the GP or health messaging on the local TV channel. Accessing a written resource from the healthcare professional, or independently at the GP surgery that they could take home and read, or ask their family to read, was requested from all groups, alongside opportunities to share experiences and talk with experts on the menopause (face-to-face or in online communities). Although visual representation in health messaging was not considered important among the South Asian women, it was important to the Black African women involved in the study. These women described how they did not understand the symptoms or see people resembling them in health messaging with these symptoms, one woman describing it as a concern only for *‘White people.’* (Black African female, 54, London), and tended to normalise/manage the symptoms they are experiencing.

The younger women (35–40 years) in the South Asian groups, who were confident speaking in English, advocated for an accessible checklist of symptoms that women could read and take with them to the doctor to clarify if their symptoms could be the menopause. They felt that a checklist would give them the confidence to discuss their experiences with the professional.

### Communicating the problem

Communicating bodily experiences was felt to be constrained by the incongruence between the language used by healthcare professionals and women. Primary care practitioners felt that a lack of awareness of the potential whole body symptom experiences surrounding the perimenopause and/or menopause could impact how women describe what they are feeling.

For example, a bilingual participant, who often consults in the first language of the woman attending, described how:

*‘*[Women from ethnic minorities may] *explain things in different ways to somebody who’s grown up being western, who’ll say, “I’ve got hot flushes,* […] *I think I’m going through ‘the change’”; they might not understand that’s what ‘the change’ can cause. Sometimes they’ll say things like, “There’s heat coming from my tummy,”* […] *or, “The pain goes up to my head.”* […] *Their symptoms just don’t always make sense, medical sense, to us* [healthcare professionals]*.’*

(PC16, Advanced Nurse Practitioner, city, North of England)

Awareness of symptoms of the perimenopause and/or menopause and health literacy may act as a barrier to communicating symptoms. This can be compounded by a lack of clinician awareness of the potentially different presentations of perimenopause and/or menopausal symptoms in different ethnic minority groups:

*‘Certainly in my patients who are maybe first or second generation Bangladeshi or Indian or Pakistani, menopause will often present as pain,* […] *it’s all, “Oh, I’m aching, I’m aching,”* […] *so it’s a very different presentation.’*

(PC27, GP, town in central England)

*‘A lot of* [women from ethnic minorities] *do present with things like aches and pains, and low mood, and a lot of it gets attributed to physical ailments and it’s quite common. A lot of them start complaining in early 30s and I think, by the time they hit the menopause age, it probably does get missed that it’s actually menopause rather than something musculoskeletal.’*

(PC40, GP, North London)

If the practitioner felt unable — through communication or time constraints — to unpick these presentations, there was a risk that women would receive suboptimal care:

*‘A good menopause consult cannot be done in 10 minutes. I mean you struggle to do it properly in 20 minutes, but you’re lucky if you get 20 minutes.’*

(PC45, GP, North London)

Participants felt that at this stage in their help-seeking journey, if the woman is unable to advocate for herself, the clinician’s knowledge and time available significantly impacts on what is achievable within the consultation, and ultimately on the patient’s perception of the quality of care experienced.

#### Reflections from the lived experience group

Hot flushes were common across the group, with one woman describing waking in the night soaked in sweat; this had been going on for 12 years but she didn’t know *‘what to ask for.’*

Other common symptoms were vulval itching, fatigue, joint pains, forgetfulness, low mood, irritability, anxiety, insomnia, bloating and dyspepsia, and headaches. Heavy vaginal bleeding and anaemia before their periods stopped were common across the groups.

Most of the women had consulted the GP about one or more of these symptoms at various times but felt no one had suggested that they might be linked to the menopause. Some of the women described being told it was normal for their age and to manage it. Others described being given medicine for depression, blood pressure (dizziness and palpitations), and thrush. Many women in the South Asian ethnic minority group were living with diabetes and were told their vulval itching, unresolved by anti-fungal pessaries, was due to their diabetes, highlighting the challenges for them (and clinicians) of unpicking symptoms in the context of multiple health concerns and possibilities.

None of these women had previously discussed the other symptoms they were experiencing, such as hot flushes, low libido, or low mood, or had been offered an examination. All the women who attended a GP with symptoms described feeling they were not listened to, and felt they had to attend multiple times, thus reducing their trust in the clinician and the health system.

Many of the women described feeling too embarrassed to discuss their symptoms, (both as part of the current study and before with friends and family as well as with the doctor or nurse), as they did not realise other women may also share these experiences. When asked which was most sensitive to talk about — periods or mental distress around menopause — the resounding answer was mental distress.

Mental distress was described as being denied within the family context and wider community, making it difficult for women to seek help. There was a prevailing attitude across the groups of ‘getting on with it,’ trying to keep busy or seeking comfort in their faith.

### Joining the dots: taking a holistic approach to care

When practitioners were able to hear and interpret the woman’s experience and propose their origin as the perimenopause and/or menopause, they found this communication of knowledge was well received:

*‘Some of the women that I’ve spoken to about* […] *the menopause, I think they found it a real revelation, it’s not that there’s something wrong with them. That it’s a natural part of life and of ageing.’*

(PC21, GP, city in central England)

However, participants felt that it takes time, often over more than one consultation, alongside knowledge of different perimenopause and/or menopause presentations by the practitioner to assess the woman holistically in light of her symptoms and age profile:

*‘I think previously women’s symptoms were dismissed as depression, or anxiety, or it’s just a cultural thing, it’s stress, it’s lots of other things, but they are coming forward, they are complaining of hot flushes and sleeplessness and anxiety symptoms, which we say to them, “Oh, it’s age-relevant, we’ve got to look at your age, this could be causing your symptoms,” and rather than treating each symptom, you’ve got to treat the root cause, and they are coming forward.’*

(PC16, Advanced Nurse Practitioner, a city in the North of England)

Despite the availability of training for practitioners in primary care, participants felt women’s health, including the perimenopause and/or menopause, rests largely with female practitioners by default as they are seen by patients to offer a more holistic approach:

*‘My male colleagues say, “We don’t feel we have the vocabulary or confidence to ask some of the questions when thinking about menopause whereas female doctors naturally do.”’*

(PC33, GP, Greater Manchester)

*‘My two male colleagues haven’t done any British Menopause Society updates so they’re not the right people to see, they can do the very basic*[s]*, but if something needs a more layered approach, they haven’t got the* — *it’s not their area of interest or skill.’*

(PC27, GP, town in central England)

Female practitioners expressed concern that if no female practitioners are available for women from ethnic minorities to consult with, this could further impact their care.

#### Reflections from the lived experience group

There was a general feeling of distrust towards medical care among the groups, despite complimenting primary healthcare staff on being *‘very good.’* (a group agreement from the Urdu speaking women).

Eight of the women said they had not felt listened to when speaking with a male clinician, even if he spoke Urdu (the Bengali women of the group were also fluent in Urdu), but access to a female clinician could be challenging. Three women specifically mentioned no one explaining the symptoms they may experience following hysterectomy. Consequently, they described not understanding when they experienced changes to their skin and hair, hot flushes, and extreme irritability that caused discord within their family. They struggled and ‘just got on with it.’ (comment made by women who have experienced hysterectomy across all three participant groups).

## DISCUSSION

### Summary

The literature shows that women from ethnic minorities may present with symptoms of menopause that are different, or described differently, from their White peers.^1^^–^5 This study has shown that there is a communication gap in the consultation, rising from a lack of awareness of perimenopause and/or menopausal symptomatology in some women from ethnic minorities that does not allow them to advocate for their health, compounded by a lack of knowledge or confidence in some primary care clinicians to interpret and connect symptom presentation with a holistic menopause care lens.

### Strengths and limitations

This study uniquely explores the communication and knowledge constraints of primary care practitioners’ in meeting the perimenopause and/or menopause needs of women from ethnic minorities in parallel to the nuanced experience, presentation, and understanding of symptomatology among a small group of women from ethnic minorities.

The principal limitation of this study is the restricted range of the public consultation (14 participants from three highly diverse communities). Existing links with two South Asian community groups and a university’s ‘Diversity in Research’ public involvement group provided a way of reaching study participants, but attempts at making new connections with community-based organisations supporting other women from ethnic minorities were unsuccessful. Although the public consultation is not reflective of the diversity of women living in the UK, it has given a small selection of women an opportunity to voice their experience and interpretation of these research findings. The dominance of female practitioners in the interview sample (40 female practitioners versus 5 male) may have impacted the nuanced sensitivity of these data to women from ethnic minorities’ experiences, and this is a complex area for further research. However, this purposive sampling approach is a strength of the methodology to achieve data saturation.

### Comparison with existing literature

The complexity and interdependence of women’s health experiences during the perimenopause and/or menopause places primary care as ideally suited to support women seeking help for the perimenopause and/or menopause because of its holistic, integrated, multimorbid expertise,^26^ and universal accessibility.^27^ However, despite a rise in perimenopause and/or menopause consultations among primary care clinicians,^28^ there is still a knowledge and confidence gap in practice. A lack of training in medical schools and reliance on self-directed education once in general practice^29^ is unsustainable in a service struggling to meet patient demand.

Furthermore, guidelines, training, and structural support for cross cultural communication consultations exist but their implementation is ad hoc.^23^ Experience of complex and inflexible service arrangements,^30^ inconsistent language support,^31^ and varying levels of health literacy among the population presenting to primary care amplify the challenges faced by women and their experiences of accessing health care.^32^

### Implications for practice

Training priorities in the pressured services of primary care polarise expertise in practice, reinforcing the gendered distribution of consultations and further deskilling of practitioners. Presentation of perimenopause and/or menopause symptoms across the population should be incorporated into core and updated primary care training as recommended by the All-Party Parliamentary Group on menopause.^33^

Both clinicians and the lived experience group of women from ethnic minorities requested educational resources in non-European languages. This would help bridge the gap by supporting the awareness raising of perimenopause and/or menopause experiences in ethnic minority communities, to inform help-seeking and communication of embodied experiences of the perimenopause and/or menopause, and as a prerequisite for decision-making about treatment options and self-care.

Given the complex nature of connecting perimenopause and/or menopausal symptoms and the inter-relationship with other chronic conditions, the availability of accurate and trusted interpreter support during the consultation is necessary for this complexity to be explored safely and thoroughly in a conversation between the woman and practitioner. The higher risks experienced by women from ethnic minorities, when compared to their White counterparts, of cardiovascular disease, osteoporosis, and diabetes, increase as a consequence of the hormonal changes of the menopause.^34^ If the communication and knowledge gap surrounding women from ethnic minorities’ experiences of the menopause can be bridged comprehensively, there is also scope to improve women’s immediate quality of life and potentially reduce future disease risk.
